# The response of soil microbial communities to variation in annual precipitation depends on soil nutritional status in an oligotrophic desert

**DOI:** 10.7717/peerj.4007

**Published:** 2017-11-09

**Authors:** Cristina Montiel-González, Yunuen Tapia-Torres, Valeria Souza, Felipe García-Oliva

**Affiliations:** 1 Instituto de Investigaciones en Ecosistemas y Sustentabilidad, Universidad Nacional Autónoma de México, Morelia, Michoacán, Mexico; 2 Escuela Nacional de Estudios Superiores Unidad Morelia, Universidad Nacional Autónoma de México, Morelia, Michoacán, Mexico; 3 Instituto de Ecología, Universidad Nacional Autónoma de México, Mexico

**Keywords:** Nutrients, Enzymes, Chihuahuan desert, Microbial physiological adjustments, Stoichiometry ratio, Threshold elemental ratio

## Abstract

**Background:**

Soil microbial communities (SMC) play a central role in the structure and function of desert ecosystems. However, the high variability of annual precipitation could results in the alteration of SMC and related biological processes depending on soil water potential. The nature of the physiological adjustments made by SMC in order to obtain energy and nutrients remains unclear under different soil resource availabilities in desert ecosystems. In order to examine this dynamic, the present study examined the effects of variation in annual precipitation on physiological adjustments by the SMC across two vegetation-soil systems of different soil organic matter input in an oligotrophic desert ecosystem.

**Methods:**

We collected soil samples in the Cuatro Ciénegas Basin (Mexico) under two vegetation covers: rosetophylous scrub (RS) and grassland (G), that differ in terms of quantity and quality of organic matter. Collections were conducted during the years 2011, 2012, 2013 and 2014, over which a noticeable variation in the annual precipitation occurred. The ecoenzymatic activity involved in the decomposition of organic matter, and the concentration of dissolved, available and microbial biomass nutrients, were determined and compared between sites and years.

**Results:**

In 2011, we observed differences in bacterial taxonomic composition between the two vegetation covers. The lowest values of dissolved, available and microbial nutrients in both cover types were found in 2012. The G soil showed higher values of dissolved and available nutrients in the wet years. Significant positive correlations were detected between precipitation and the ratios Cmic:Nmic and Cmic:Pmic in the RS soil and Cmic:Pmic and Nmic:Pmic in the G soil. The slopes of the regression with Cmic and Nmic were higher in the G soil and lower in the RS soil. Moreover, the SMC under each vegetation cover were co-limited by different nutrients and responded to the sum of water stress and nutrient limitation.

**Discussion:**

Soil community within both sites (RS and G) may be vulnerable to drought. However, the community of the site with lower resources (RS) is well adapted to acquire P resources by ecoenzyme upregulation during years with adequate precipitation, suggesting that this community is resilient after drought occurs. Under the Global Climate Change scenarios for desert ecosystems that predict reduced annual precipitation and an increased intensity and frequency of torrential rains and drought events, the soil microbial communities of both sites could be vulnerable to drought through C and P co-limitation and reallocation of resources to physiological acclimatization strategies in order to survive.

## Introduction

In desert ecosystems, precipitation is highly variable among years and this variability has increased in recent years due to the effect of Global Climate Change (GCC) (Bell et al., 2014; IPCC, 2013). The scenarios derived from GCC models for desert ecosystems predict reduced annual precipitation, as well as increases in the annual precipitation variability by the end of the 21st century, including an increase in the frequency and intensity of both torrential rain and drought events (Holmgren et al., 2006; IPCC, 2013). The high variability of annual precipitation projected for desert ecosystems could alter biological processes dependent on soil water potential, as is the case with the processes related to soil organic matter (SOM) decomposition (D’Odorico & Bhattachan, 2012; Fay et al., 2008; Thomey et al., 2011). For example, enzymatic activity stimulated by rainfall in desert ecosystems may result in most of the total annual mineralization that occurs in desert soils (Manzoni, Schimel & Porporato, 2012). However when soil water potential decreases, the metabolic activity of most soil microbial species is reduced, and thus a decline in nutrient mineralization can occur. Additionally, soil drying reduces enzymatic activity and microbial mobility, which reduces substrate supply for the decomposers (Henry, 2013; Manzoni, Schimel & Porporato, 2012). Likewise, studies in a semiarid region in New Mexico (Cregger et al., 2012) and in the Chihuahuan Desert (Bell et al., 2009; Bell et al., 2014) showed that the high precipitation variability significantly altered the structure of the soil microbial community, mainly due to a change in the fungal/bacterial ratio and consequently altered microbial community functional dynamics.

Microbial communities play a central role in the structure and functioning of desert ecosystems since they represent an important pool of soil C, N, and P. Indeed, it has been suggested that the amount of N and P contained within the soil microorganism biomass is comparable to the N and P content within the plant biomass in desert ecosystems (Coleman & Whitman, 2005). Moreover, microbial communities can help accelerate the transformation of molecules containing C, N, and P by producing soil extracellular enzymes (ecoenzymes) (Sinsabaugh & Follstad Shah, 2012; Sinsabaugh, Hill & Shah, 2009) that lead to the fragmentation, depolymerization and mineralization of organic matter (Singh et al., 2014). Microorganisms can only assimilate soluble organic compounds of a molecular weight lower than 1 kDa and must therefore break down, or depolymerize, most of the organic matter molecules (where between 72 and 87% of the DOC in grassland soils is larger than 1 kDa) in order to access the nutrients and energy contained within the organic molecules (Cregger et al., 2012; Farrell et al., 2014; Jones et al., 2012). The microorganisms produce hydrolytic or oxidative ecoenzymes that degrade organic matter, producing assimilable dissolved organic nutrients that are rapidly immobilized within their biomass (Conant et al., 2011; Sinsabaugh & Follstad Shah, 2012). Additionally, in desert ecosystems, the natural distribution of different vegetation types can produce spatial heterogeneity in the quantity and quality of organic matter (Austin et al., 2004; Housman et al., 2007). In these ecosystems, the depolymerization process will therefore require the production of different ecoenzymes, since the organic matter under each vegetation type contains a particular combination of structurally simple and complex molecules that promote differences in the soil nutrient dynamics mediated by the microbial community (Conant et al., 2011). However, the complete organic matter decomposition process requires a chain of enzymatic reactions where each ecoenzyme acts on a different substrate and is produced by different microbial groups (Ekschmitt et al., 2005). Additionally, the soil microbial communities can exhibit functional redundancy in the ecoenzyme production (Allison & Martiny, 2008).

Soil microorganisms have developed mechanisms of physiological acclimatization to cope with precipitation variability (Schimel & Schaeffer, 2012). These mechanisms generate physiological costs for the microbial community that derive from the need for high investments of energy (C) and nutrients (N and P) in order to survive (Classen et al., 2015; Schimel, Balser & Wallenstein, 2007; Schimel & Schaeffer, 2012). This high demand for energy (C) and nutrients (N and P) can be offset by reallocation of these resources, generating a trade-off in which the microbial community invests C, N and P in either growth or survival (Evans & Wallenstein, 2012; Schimel, Balser & Wallenstein, 2007). Some consequences of such resource redirection are: (1) a limited production of ecoenzymes for nutrient acquisition (i.e., for SOM decomposition) (Burns et al., 2013; Henry, 2013; Steinweg et al., 2013) and (2) reduced growth of the microbial community (i.e., decreased protein synthesis) (Schimel, Balser & Wallenstein, 2007). Resource reallocation increases the vulnerability of some microbial groups that produce a change in the structure and function of the soil microbial community also affecting the energy flow (C) and nutrient dynamics of N and P at the ecosystem level (Esch, Lipson & Cleland, 2017; Evans & Wallenstein, 2012; Thibault & Brown, 2008). This variability strongly affects microbial community development in resource-limited environments, because the adaptation rates of microbial species are constrained by the resource cost of physiological adjustment (Wallenstein & Hall, 2012). Wallenstein & Hall (2012) proposed that sites limited by nutrients are more vulnerable to annual rainfall variability, because the microbial community must invest energy in nutrient acquisition, and consequently reducing its capacity for adaptation required by fluctuation in water availability. Sites with low resource availability could be therefore more vulnerable to annual precipitation variability.

The Chihuahuan desert has been classified as one of the most biologically outstanding habitats globally by the World Wildlife Fund (Archer & Predick, 2008). The Cuatro Ciénegas Basin (CCB), which is the study site of the present investigation, is part of the Chihuahuan desert and is considered the most important wetland of Mexico for its high levels of endemism and biodiversity (Souza et al., 2011). Moreover, the CCB has been listed as an ultra-oligotrophic site due to low P concentrations in the water and soil, which can constitute a strong potential for P limitation of microbial growth (Elser et al., 2005; Tapia-Torres et al., 2015a). A study in the CCB desert reported that, in the same soil type with different vegetation cover (grassland and desert scrub) differences in OM content promotes variation in DOC concentration, which represents the main energy source for soil microorganisms (Tapia-Torres et al., 2015b). The higher DOC concentration under grassland soil compared to desert scrub soil favored a higher microbial N immobilization and a higher C availability, therefore significantly reducing soil N losses (Tapia-Torres et al., 2015b). Another study in the CCB that compared two sites with different soil moisture content showed that the site with the highest moisture content and concentration of DOC also exhibited higher NH${}_{4}^{+}$, microbial C and N concentrations, and also presented higher diversity, richness and evenness of soil bacterial community compared to the dry site (López-Lozano et al., 2012). Both studies suggest that differences in DOC concentration (energy availability) and microbial community composition promoted different nutrient dynamics. In the sites with organic matter providing lower DOC concentrations, the microbial communities may be co-limited by energy and nutrients and yet they must invest more energy in order to obtain the most limiting nutrients. An indicator that helps us understand how resources are reallocated by the microbial community to cope with the nutrient limitation is the combination of: (1) the stoichiometry ratios of C:N:P in the soil and microbial biomass (Cleveland & Liptzin, 2007) and (2) the Threshold Elemental Ratio (TER) (Sinsabaugh & Follstad Shah, 2012; Tapia-Torres et al., 2015a), which defines the element ratio at which growth is affected by nutrient limitation (represented by N and P, at high C:N or C:P) and by energy limitation (represented by C, at low C:N or C:P) (Frost et al., 2006; Sterner & Elser, 2002). The combination of stoichiometry ratios and TER indicate how resources are reallocated towards enzyme activity depending on the availability of energy (C) and nutrients (N and P) in the soil. This microbial co-limitation between energy and nutrient acquisition was also found in CCB by comparing the TER_C:N_and TER_C:P_ from two sites with the same vegetation cover (grassland), but different soil moisture and DOC availability values (Tapia-Torres et al., 2015a). The microbial communities were co-limited by C and N in the site with higher water and C availability (Churince) and were co-limited by C and P in the site with lower water and C availability (Pozas Azules). In addition, these authors argue that this limitation favors an elevated allocation of N-acquisition enzymes relative to energy/C enzymes in Churince, while for Pozas Azules, an elevated investment in ecoenzymes of P acquisition is found (Tapia-Torres et al., 2015a). These results support the notion that soil microbial communities can adjust their metabolism by allocating more resources (i.e., energy and production of ecoenzymes) to the accumulation of scarcer nutrients, and fewer resources to the acquisition of abundant nutrients. The ratios of C:N:P in microbial biomass are therefore constrained relative to nutrient (Cleveland & Liptzin, 2007) and energy availability. These studies suggest that both vegetation and soil moisture content may determine differences in: (1) soil nutrient dynamics, (2) the diversity of the soil microbial community and (3) the C:N:P ratios of the microbial biomass in this ecosystem.

To date, the physiological adjustments made by the soil microbial communities under different soil resource availability in order to obtain energy and nutrients in desert ecosystems with high precipitation variability remain unclear. To elucidate this dynamic, the present study examined the effects of rainfall variation on the physiological adjustments made in order to obtain energy and nutrients by the soil microbial community from two vegetation-soil systems with different soil organic matter inputs in an oligotrophic desert ecosystem. Our hypothesis is that, in a site with high soil resources availability, the soil microbial communities invest less energy in the acquisition of nutrients (i.e., ecoenzymatic production), favoring nutrient accumulations within the biomass (i.e., immobilization). Our predictions are: (1) in a site that presents low soil nutrient availability (rosetophylous scrub—RS), the soil microbial community will invest more energy in the production of ecoenzymes in order to depolymerize and mineralize, thus favoring nutrient availability; while in a site with high soil nutrient availability (grassland—G), the soil microbial community will invest more energy in biomass growth; and (2) in the site with greater soil resources availability (G), the microbial community will be less vulnerable to changes in precipitation. To test the hypothesis, we collected soil samples in the CCB from sites under two vegetation covers (RS and G) that differ in terms of the quantity and quality of the organic matter present. Collections were conducted during years: 2011 (February), 2012, 2013 and 2014 (September), over which a noticeable variation in annual precipitation took place. The ecoenzyme activity involved in the decomposition of organic matter, as well as the concentration of dissolved, available and microbial biomass nutrient, were determined and compared between sites and years. With the ecoenzymatic and biogeochemistry data we calculated the TER_C:nutrient_, SEA, the nutrient ratios and performed regressions between the precipitation and the concentrations and ratios of C, N and P in microbial biomass.

## Material and Methods

### Study site

The study was carried out in the Cuatro Ciénegas Basin (CCB; 26°45′-27°00′N and 101°48′-102°17′W) in central northern Mexico, within the Chihuahuan Desert. The CCB has an area of 150,000 km^2^, with an elevation of 740 m.a.s.l. The climate is arid with an average annual temperature of 21 °C and 252 mm of annual rainfall, which is concentrated during the summer months (http://smn.cna.gob.mx/). However in the last 30 years the annual precipitation showed a high variability among years. In this study the annual precipitation was estimated as the amount of rain accumulated 9-months before the sampling month. The precipitation data were obtained from meteorological station 5044 “Cuatro Cienegas” located at 26°59′0″N and 101°04′0″W (http://smn.cna.gob.mx/). Annual precipitation and the average temperature of the sampling months varied strongly during the four studied years: the year 2011 was the wettest year (348 mm and 25 °C), 2012 was particularly dry and hot (89 mm and 28 °C) and was followed by two wet years (217 mm and 230 mm for 2013 and 2014, respectively) with lower temperatures (24.9 and 24.8 °C for 2013 and 2014, respectively).

Jurassic-era gypsum is the dominant parent material on the western side of the basin (McKee, Jones & Long, 1990). According to the WRB classification (2007), the predominant soil on the western side of the basin is *Gypsisol*. The main vegetation types are: (1) grassland (G), dominated by *Sporobolus airoides* (Torr.) Torr. and *Allenrolfea occidentalis* (S. Watson) Kuntze; (2) microphyll scrub, dominated by *Jatropha dioica* Cerv., *Larrea tridentate* (DC) Cov. and *Fouqueria sp* Kunth (Perroni, García-Oliva & Souza, 2014); and (3) rosetophylous scrub (RS) dominated by *Dhasylirium cedrosanum* Trel.*,* and *Yucca treculeana* Carriére (González, 2012).

### Sampling

Mean air temperature for the sampling month (September) and annual rainfall data in each studied year were obtained from the meteorological station “Rancho Pozas Azules” INIFAP. Soil collection was carried out in Churince on the west side of the CCB, where *Gypsisol* is the predominant soil type (Perroni et al., 2014). The samples were taken from two vegetation cover types, rosetophylous scrub (RS) and grassland (G), during February (2011) and September (rainy of 2012, 2013 and 2014). For each vegetation cover, we sampled seven sites located at a distance of 140 m apart, along a one km north-to-south transect. At each sampling site, a 4 × 4 m plot was demarcated and five soil samples were taken from the first 15 cm of soil depth within the plot, and mixed to produce one compound sample per site. A total of seven composite samples were therefore obtained from each vegetation cover in each sampling year. The soil samples were stored in black plastic bags at 4 °C until subsequent laboratory analysis.

### Moisture and pH

Soil pH was measured in deionized water (soil/solution, 1:2 w:v) with a digital pH meter (Corning™). A subsample of 100 g was oven-dried at 75 °C to constant weight for soil moisture determination using the gravimetric method.

### Biogeochemical analyses

#### Nutrient analysis

All Carbon (C) forms analyzed were determined with a Total Carbon Analyzer (UIC Mod. CM5012; Chicago, USA), while nitrogen (N) and phosphorus (P) concentrations were determined by colorimetric analyses, using a Bran Luebbe Auto Analyzer III (Norderstedt, Germany). Microbial P and enzymatic activity were determined by colorimetric analyses using a spectrophotometer Evolution 201 (Thermo Scientific Inc.).

#### Total nutrients

Prior to analysis of total nutrient forms, soil samples were dried and milled with a pestle and agate mortar. Total C (TC) and inorganic C (IC) were determined by combustion and coulometric detection (Huffman, 1977). Organic total C (OTC) was calculated as the difference between TC and IC. For total N (TN) and total P (TP) determination, the samples were digested in a mixture of concentrated H_2_SO_4_, H_2_O_2_ (30%) and K_2_SO_4_ plus CuSO_4_, the latter acting as a catalyst at 360 °C. Nitrogen was determined by the macro Kjeldahl method (Bremmer, 1996), while P was determined by the molybdate colorimetric method, following ascorbic acid reduction (Murphy & Riley, 1962).

#### Dissolved and available nutrients and those within the microbial biomass

The dissolved, available and microbial nutrient forms were extracted from fresh field soil samples. Dissolved nutrients were extracted from 20 g of soil with deionized water after shaking for 45 min and then filtering through a Whatman No. 42 and a 0.45 µm nitrocellulose membrane (Jones & Willett, 2006). The filtrate was used to determine the total dissolved C (TDC), as measured with an Auto Analyzer of carbon (TOC CM 5012) module for liquids (UIC-COULOMETRICS). Inorganic dissolved C (IDC) was determined in an acidification module CM5130. One aliquot of the filtrate was used to determine ammonium (DNH${}_{4}^{+}$) and dissolved inorganic P (DIP) in a deionized water extract. Total dissolved N and P (TDN and TDP, respectively) were digested in a mixture of concentrated H_2_SO_4_, H_2_O_2_ (30%) at 250 °C. Nitrogen was determined by the macro Kjeldahl method (Bremmer, 1996), while P was determined by the molybdate colorimetric method, following ascorbic acid reduction (Murphy & Riley, 1962). Dissolved organic C, N and P (DOC, DON and DOP respectively) values were calculated as the difference between the total dissolved forms and the inorganic dissolved forms.

Available inorganic nitrogen forms (NH${}_{4}^{+}$ and NO${}_{3}^{-}$) were extracted from 10 g of soil with 2M KCl, followed by filtration through a Whatman No. 1 paper filter, and determined colorimetrically by the phenol-hypochlorite method (Technicon, 1977). Available inorganic phosphorous (Pi) was extracted with 0.5 M NaHCO_3_, pH 8.5 (Tiessen & Moir, 2008) and determined colorimetrically using the molybdate-ascorbic acid method (Murphy & Riley, 1962).

Carbon (Cmic) and N (Nmic) concentrations within the microbial biomass were determined from 20 g of soil by the chloroform fumigation extraction method (Vance, Brookes & Jenkinson, 1987). Fumigated and non-fumigated samples were incubated for 24 h at 25 °C and constant relative humidity. Cmic and Nmic were extracted from fumigated and non-fumigated samples with 0.5 MK_2_SO_4_, filtered through a 0.45 µm nitrocellulose membrane (Brookes, Powlson & Jenkinson, 1984). Carbon concentration was measured from each extract, as the total (TC) and inorganic (IC) carbon contents, using the method described before. The difference between TC and IC was used for Cmic calculation. To determine the Nmic concentration one aliquot of the filtrate extracted was acid digested and determined as TN by Macro-Kjeldahl method (Brookes, Powlson & Jenkinson, 1984). Phosphorus within microbial biomass (Pmic) was extracted from 5 g of soil by the chloroform fumigation extraction and incubation method (Vance, Brookes & Jenkinson, 1987). Pmic was extracted using NaCO_3_ 0.5M, pH 8.5 and digested in a mixture of H_2_SO_4_ 11N and (NH_4_)_2_S_2_O_8_ at 50%, with the latter acting as a catalyst at 120 °C (Lajtha et al., 1999). Pmic was determined colorimetrically by the molybdate-ascorbic acid method (Murphy & Riley, 1962). The values of Cmic, Nmic and Pmic were calculated as the difference between fumigated and non-fumigated samples using correction factors of K_EC_ 0.45 (Joergensen, 1996), K_EN_ 0.54 (Joergensen & Mueller, 1996) and K_P_ 0.4 (Lajtha et al., 1999) for Cmic, Nmic and Pmic, respectively. Finally, the values of Cmic, Nmic and Pmic were corrected to a dry soil basis.

### Molecular analysis

Bacterial composition analysis was performed on the samples from the wettest year (2011). We extracted DNA from each soil sample using the methodology described in López-Lozano et al. (2013) and sent it to J. Craig Venter Institute (JCVI) in order to construct a 16S library using 454 ROCHE tag, 50,000 reads per site of 500 bp and primers 341F-926R. Sequences were trimmed and chimeras eliminated using JCVI protocols. Taxa were assigned using Blast via JCVI pipeline, these methods are detailed by Tanenbaum et al. (2010).

### Ecoenzyme activity analyses

The activities of six ecoenzymes (extracellular enzymes) involved in the cleavage of organic molecules with C, N and P were measured: *β*-1,4-glucosidase (BG), cellobiohydrolase (CBH), *β*-1,4-N-acetylglucosaminidase (NAG), polyphenol oxidase (PPO), phosphomonoesterase (PME) and phosphodiesterase (PDE), using assay techniques reported by Tabatabai & Bremner (1969), Eivazi & Tabatabai (1977), Eivazi & Tabatabai (1988), Verchot & Borelli (2005) and Johannes & Majcherczyk (2000).

For all ecoenzymes, we used 2 g of fresh soil and 30 ml of modified universal buffer (MUB) at pH 9 for ecoenzyme extraction. Three replicates and two control samples (soil extract with no substrate, and pure MUB with substrate) were included per assay. All ecoenzyme assays were incubated at 40 °C: the BG and CBH for 2 h, NAG for 3 h, PPO for 2.5 h, PME and PDE 1.25 h. Following the incubation period, the tubes were centrifuged at 10,000 rpm for 2 min and 750 µl of supernatant was recovered.

For all ecoenzymes with substrates containing p-nitrophenol (pNP), we diluted the supernatant in 2 ml of deionized water with 75 µl of NaOH and measured the absorbance of pNP liberated at 410 nm on an Evolution 201 spectrophotometer (Thermo Scientific Inc.). For the PPO, we used 2,2′-Azinobis [3-ethylbenzothiazoline-6-sulfonic acid]-diammonium salt (ABTS) as a substrate. The resulting supernatant was measured directly at 410 nm. Ecoenzyme activities were expressed as nanomoles of pNP per gram of dry soil per hour (nmol pNP [g SDE]^−1^ h^−1^) for substrates containing p-nitrophenol (pNP) and O_2_ formed per gram of dry soil per hour (nmolO_2_ [g SDE]^−1^ h^−1^) for the PPO, respectively. Specific enzymatic activity was calculated using Eqs. (1)–(3) (Chavez-Vergara et al., 2014; Waldrop, Balser & Firestone, 2000):

(1) \begin{eqnarray*}& & \mathrm{SEA} \mathrm{\mu }\mathrm{mol}/(\mathrm{mg}{C}_{\mathrm{mic}}\mathrm{h})=A/({\mathrm{C}}_{\mathrm{mic}}\times 0.001)\end{eqnarray*}

(2) \begin{eqnarray*}& & \mathrm{SEA} \mathrm{\mu }\mathrm{mol}/(\mathrm{mg}{N}_{\mathrm{mic}}\mathrm{h})=B/({\mathrm{N}}_{\mathrm{mic}}\times 0.001)\end{eqnarray*}

(3) \begin{eqnarray*}& & \mathrm{SEA} \mathrm{\mu }\mathrm{mol}/(\mathrm{mg}{\mathrm{P}}_{\mathrm{mic}}\mathrm{h})=C/({\mathrm{P}}_{\mathrm{mic}}\times 0.001)\end{eqnarray*}

where *A* is the enzymatic activity of BG or CBH or PPO, *B* is the enzymatic activity of NAG and *C* is the enzymatic activity of PME or PDE.

### Data analysis

#### Biogeochemistry and ecoenzymatic analysis

Soil biogeochemistry and ecoenzymatic data were subjected to a repeated measures analysis of variance (RMANOVA) (Von Ende, 2001). Vegetation cover types (RS and G) were considered as a between-subject factor and year (2012, 2013 and 2014), and their interaction, were considered as within-subject factors. When RMANOVA indicated significant factor effects, mean comparisons were performed with Tukey’s multiple comparisons test (Von Ende, 2001). Ecoenzyme activities were normalized to units per μg of available organic carbon (OC) using the DOC data corresponding to each sample (Tapia-Torres et al., 2015a). Data were log_e_-transformed prior to regression analysis in order to conform to the conventions of stoichiometric analyses and to normalize variance (Sinsabaugh & Follstad Shah, 2012; Sterner & Elser, 2002). After that, relationships between ecoenzyme activities were calculated with a type II regression, using SMATR (R Development Core Team, 2007).

To detect the relationship between precipitation and nutrients immobilized by microbial biomass, we applied two simple regression analyses using the annual accumulated precipitation prior to the sampling date with: (1) nutrient concentration within the microbial biomass (Cmic, Nmic and Pmic) and (2) the microbial biomass nutrient ratios (Cmic:Nmic, Cmic:Pmic and Nmic:Pmic). The data used in the regression analyses corresponded to the years 2011, 2012, 2013 and 2014.

#### Stoichiometric analyses and threshold elemental ratio

We calculated the degree of soil community-level microbial C:N and C:P homeostasis by calculating the slope of log_e_ C:N_R_ (resources) versus log_e_ C:N_*B*_ (microbial biomass) or the slope of log_e_ C:P_R_ versus log_e_ C:P_*B*_ scatterplot (Sterner & Elser, 2002). Moreover, we followed Sinsabaugh, Hill & Shah (2009) in order to calculate the TER for C:N and C:P to relate the measured ecoenzyme activity with Ecological Stoichiometry Theory (EST) and the Metabolic Theory of Ecology (MTE), using Eqs. (4) and (5):

(4) \begin{eqnarray*}& & {\mathrm{TER}}_{\mathrm{C:N}}=((\mathrm{BG}/\mathrm{NAG}){B}_{\mathrm{C:N}})/{n}_{0}\end{eqnarray*}

(5) \begin{eqnarray*}& & {\mathrm{TER}}_{\mathrm{C:P}}=((\mathrm{BG}/\mathrm{PME}){B}_{\mathrm{C:P}})/{p}_{0}\end{eqnarray*}

where TER_C:N_ and TER_C:P_ are the threshold ratios (dimensionless), BG/NAG is the ecoenzymatic activity ratio for *β*-1,4-glucosidase and *β*-1,4-N-acetylglucosaminidase, BG/PME is the ecoenzymatic ratio for *β*-1,4-glucosidase and phosphomonoesterase, *B*_C:N_ and *B*_C:P_ are the C:N or C:P ratios of the microbial biomass (respectively) and *n*_0_ and *p*_0_ are the dimensionless normalization constants for N and *P*, respectively. These normalization constants *p*_0_ and *n*_0_ are the intercepts in the SMA regressions for log_e_ (BG) *vs.* log_e_ (NAG) and log_e_ (BG) *vs.* log_e_ (PME) respectively (Tapia-Torres et al., 2015a). For a more detailed analysis of the derivation of the equations, see Sinsabaugh, Hill & Shah (2009).

## Results

### Soil moisture, and pH

Regardless of vegetation cover, soil moisture was higher in 2013 and 2014 than in 2012; while the G soil had higher soil moisture than the RS soil, regardless of year (Tables 1 and 2). In the driest year (2012), soil pH was higher than in the wetter years (2013 and 2014), with an exception in the G soil in 2014 (Tables 1 and 2). Soil pH correlated with annual precipitation in both sites (*R*^2^ =  − 0.85 and *R*^2^ =  − 0.61 for RS and G, respectively), as well as soil moisture correlated with annual precipitation (*R*^2^ = 0.76 and *R*^2^ = 0.88 for RS and G, respectively).

**Table 1:** Means and (standard errors) of soil nutrients and ratios in the rosetophylous scrub (RS) and grassland (G) soils over three consecutive years (2012, 2013 and 2014) in the Cuatro Ciénegas Basin, Coahuila, Mexico. Different uppercase letters (A and B) indicate significantly different means (*P* < 0.05) between vegetation cover types (rosetophylous scrub and grassland) within the same sampling year (2012, 2013 and 2014); whereas different lowercase letters (a, b and c) indicate significantly different means (*P* < 0.05) among sampling dates within the same site.

	Year					
	2012		2013		2014	
	RS	G	RS	G	RS	G
Moisture (%)	12.7 (1.1)^Bc^	24.6 (2.5)^Ab^	24.6 (3)^Bab^	43.5 (1.3)^Aa^	16.4 (1.0)^Bb^	37.1 (7.1)^Aa^
pH	8.5 (0.06)^Aa^	8.3 (0.04)^Ba^	8.1 (0.03)^Ab^	8.1 (0.02)^Ab^	8.1 (0.02)^Ab^	8.1 (0.1)^Aab^
Dissolved organic nutrient concentration						
DOC (µg g^−1^)	9 (1)^Ab^	19 (4)^Ac^	23 (4)^Ba^	52 (1)^Ab^	28 (2)^Ba^	67 (4)^Aa^
DON (µg g^−1^)	4.1 (0.5)^Bb^	7.0 (0.6)^Ab^	5.5 (0.5)^Bab^	10.8 (0.8)^Aa^	6.9 (0.3)^Ba^	7.8 (0.4)^Aab^
DOP (µg g^−1^)	1.2 (0.2)^Ab^	0.3 (0.3)^Ab^	2.8 (0.2)^Ba^	5.1 (0.2)^Aa^	2.8 (0.2)^Ba^	5.3 (0.5)^Aa^
DOC:DON	2.3 (0.6)	3.1 (0.6)	4.2 (0.6)	4.9 (0.6)	4.0 (0.6)	6.8 (0.6)
DOC:DOP	7.9 (3.6)	15.3 (3.6)	8.2 (1.3)	18.8 (1.3)	10.3 (1.2)	13.0 (1.1)
DON:DOP	3.6 (0.4)	6.8 (3.3)	2.0 (0.1)	2.1 (0.1)	2.6 (0.4)	1.5 (0.1)
Available nutrient concentration						
NH${}_{4}^{+}$ (µg g^−1^)	2.8 (0.2)^Ba^	6.3 (0.5)^Ac^	3.6 (0.2)^Ba^	11.8 (1.1)^Aa^	2.7 (0.4)^Ba^	8. 9 (0.2)^Ab^
NO${}_{3}^{-}$ (µg g^−1^)	10.4 (1.4)^Aa^	6.7 (1.4)^Ba^	1.7 (0.3)^Ab^	3.2 (0.4)^Aab^	1.7 (0.1)^Ab^	1.0 (0.1)^Ab^
Pi (µg g^−1^)	1.9 (0.2)	2.5 (0.2)	2.9 (0.4)	4.5 (0.4)	3.9 (0.6)	5.3 (0.6)

**Notes.**

DOC dissolved organic carbon DON dissolved organic nitrogen DOP dissolved organic phosphorus NH${}_{4}^{+}$ available ammonium NO${}_{3}^{-}$ available nitrate Pi Available inorganic phosphorus

**Table 2:** F-ratios and significant levels of the repeated-measures ANOVA for soil variables quantified in the rosetophylous scrub and grassland soils over three consecutive years (2012, 2013 and 2014) in Cuatro Ciénegas Basin, Coahuila Mexico.

Parameters	Source of variation		
	Between subjects	Within subjects	
	Vegetation cover	Year	Vegetation cover X Year
Moisture	90.7 (<0.0001)	49.1 (<0.0001)	2.7 (0.08)
pH	7.3 (0.02)	28.0 (<0.0001)	5.4 (0.01)
Dissolved nutrients			
DOC	102.1 (<0.0001)	79.2 (<0.0001)	14.5 (<0.0001)
DON	38.5 (<0.0001)	25.1 (<0.0001)	3.8 (0.03)
DOP	14.1 (0.002)	55.0 (<0.0001)	13.2 (0.0001)
DOC:DON	6.4 (0.02)	11.6 (0.0002)	2.0 (0.1)
DOC:DOP	9.1 (0.01)	0.5 (0.6)	1.8 (0.2)
DON:DOP	1.8 (0.2)	3.0 (0.07)	1.2 (0.3)
Available nutrients			
NH${}_{4}^{+}$	236.8 (<0.0001)	19.0 (<0.000)	10.5 (0.0005)
NO${}_{3}^{-}$	1.8 (0.1)	47 (<0.0001)	5.4 (0.01)
Pi	14.2 (0.003)	12.9 (0.002)	1.1 (0.3)

**Notes.**

DOC dissolved organic carbon DON dissolved organic nitrogen DOP dissolved organic phosphorus NH${}_{4}^{+}$ available ammonium NO${}_{3}^{-}$ available nitrate Pi Available inorganic phosphorus

### Dissolved organic nutrients and available nutrients

For the two vegetation covers, the lowest values of DOC, DON and DOP were found in 2012 (Table 1). In this year, the RS and the G soils had similar DOC and DOP concentrations, while the G soil had a higher DON concentration than the scrub soil; moreover, the G soil had higher dissolved organic nutrient concentrations than in the RS soil in both 2013 and 2014 (Tables 1 and 2). Consequently, the DOC:DON ratio was lower in 2012 than in the other two years (2013 and 2014) and the RS soil had lower values than the G soils (3.5 and 4.9, respectively); the RS soil also had lower DOC:DOP ratios than the G soil (9 and 16, respectively).

The year trends of available NH${}_{4}^{+}$ concentration differed between the two vegetation cover types. Available NH${}_{4}^{+}$ concentration was similar over the three years in the RS soil, while G soil samples from 2012 and 2013 had the lowest and the highest NH${}_{4}^{+}$ concentrations, respectively (Tables 1 and 2). However, the G soil had higher values than the RS soil in the three studied years. In contrast, the NO${}_{3}^{-}$ concentration was higher in the samples collected in 2012 than those of the other two years, while the RS soil had higher NO${}_{3}^{-}$ concentration than the G soil only in the 2012 samples (Tables 1 and 2). The 2012 samples had lower available P concentration than in those collected in the other two years, and the G samples had 40% higher available P concentration than the RS samples, regardless of the sampling year (4.1 and 2.9 µg P g^−1^, respectively).

### Microbial nutrients and ecoenzymatic activities

The highest and the lowest values of Cmic and Nmic were found in 2014 and 2012, respectively, and Cmic values of the G soil samples were 39% higher than in the RS soil samples, regardless of sampling year (254 and 184 µg C g^−1^, respectively). This was also the case with the Nmic and Pmic concentrations, with an exception in the 2012 samples (Tables 3 and 4). In contrast, Pmic concentrations presented no differences among years within the RS samples, while the 2012 samples had lower Pmic values than was the case in the other two years, within the G samples (Tables 3 and 4). The 2014 samples had lower Cmic:Nmic than the other two years regardless of vegetation cover type (2012 and 2013), while the lowest and the highest Cmic:Pmic and Nmic:Pmic ratios were found in 2012 and 2014, respectively (Tables 3 and 4). The RS soil samples had higher Cmic:Pmic and Nmic:Pmic ratios than in the G soil samples, with an exception in the 2012 samples (Tables 3 and 4).

**Table 3:** Means and (standard errors) of microbial biomass nutrients, and microbial nutrient ratios in the rosetophylous scrub (RS) and the grassland (G) soils over three consecutive years (2012, 2013 and 2014) in the Cuatro Ciénegas Basin, Coahuila, Mexico. Different uppercase letter (A and B) indicate that means differ significantly (*P* < 0.05) between vegetation cover types (RS and G) within the same sampling year (2012, 2013 and 2014); whereas different lowercase letters (a, b and c) indicate significantly different means (*P* < 0.05) among sampling dates within the same site.

	Year					
	2012		2013		2014	
	RS	G	RS	G	RS	G
Nutrients concentration within microbial biomass						
Cmic (µg g^−1^)	68 (12)	93 (12)	191 (20)	289 (20)	287 (16)	379 (1)
Nmic (µg g^−1^)	4.2 (0.6)^Ab^	6.4 (0.6)^Ac^	10.0 (1.0)^Bb^	22.0 (2.8)^Ab^	42.2 (1.7)^Ba^	59.8 (1.9)^Aa^
Pmic (µg g^−1^)	2.3 (0.6)^Aa^	2.5 (1.3)^Ab^	2.4 (0.1)^Ba^	6.4 (0.6)^Aa^	2.2 (0.02)^Ba^	6.1 (0.4)^Aa^
Cmic:Nmic	20 (4)	15 (4)	20 (2)	14 (2)	7 (0.3)	6 (0.3)
Cmic:Pmic	17 (5)^Ac^	9 (5)^Ac^	79 (6)^Ab^	48 (6)^Bb^	127 (0.2)^Aa^	63 (4)^Ba^
Nmic:Pmic	0.9 (0.3)^Ac^	0.4 (0.2)^Ac^	4.2 (0.4)^Ab^	3.6 (0.6)^Ab^	18.7 (0.8)^Aa^	10.1 (0.7)^Ba^

**Notes.**

Cmic microbial carbon Nmic microbial nitrogen Pmic microbial phosphorus

**Table 4:** F-ratios and significant levels of the repeated measures ANOVA for microbial nutrient concentration, microbial nutrient ratios and specific enzymatic activity quantified in the rosetophylous scrub (RS) and the grassland (G) soils over three consecutive years (2012, 2013 and 2014) in Cuatro Ciénegas Basin, Coahuila Mexico.

Parameters	Source of variation		
	Between subject	Within subjects	
	Vegetation cover	Year	Vegetation cover X Year
Dissolved nutrients			
Cmic	62.1 (<0.0001)	93.3 (<0.0001)	2.3 (0.11)
Nmic	48.7 (<0.0001)	484 (<0.0001)	12.9 (0.0001)
Pmic	24.6 (0.0003)	5.8 (0.008)	5.7 (0.009)
Cmic:Nmic	4.0 (0.07)	12.3 (0.0002)	0.7 (0.5)
Cmic:Pmic	107 (<0.0001)	92 (<0.0001)	11 (0.0005)
Nmic:Pmic	42 (<0.0001)	316 (<0.0001)	34 (<0.0001)
Specific enzymatic activity			
BG	1.2 (0.28)	22.8 (<0.0001)	1.1 (0.33)
CBH	3 (0.1)	9.9 (<0.0001)	0.2 (0.7)
NAG	8.1 (0.01)	52 (<0.0001)	10.8 (<0.0001)
PPO	8.8 (0.011)	34 (<0.0001)	4 (0.03)
PME	137 (<0.0001)	444 (<0.0001)	80 (<0.0001)
PDE	67 (<0.0001)	232 (<0.0001)	19 (<0.0001)

**Notes.**

Cmic microbial carbon Nmic microbial nitrogen Pmic microbial phosphorus BG β-1,4-glucosidase CBH cellobiohydrolase NAG β-1,4-N-acetylglucosaminidase PPO polyphenol oxidase PME phosphomonoesterase PDE phosphodiesterase

**Figure 1:**
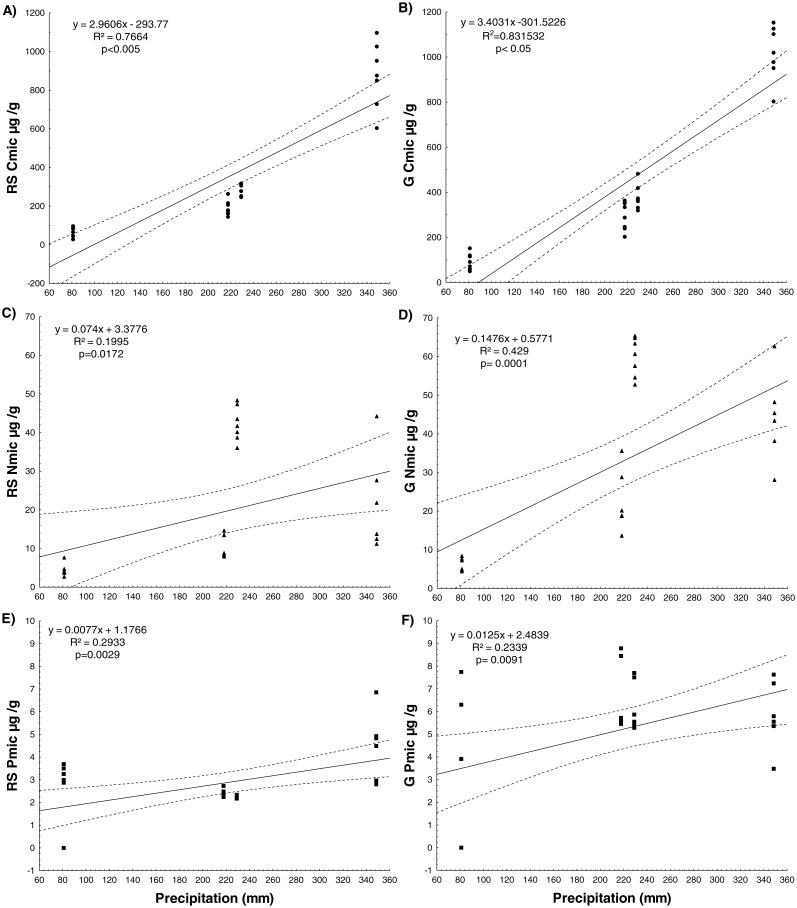
Simple linear regressions between annual accumulated precipitation before the sampling date for four years and nutrients immobilized by microbial biomass for RS soil and G soil. The dotted line represents the standard deviation at 0.95.

**Figure 2:**
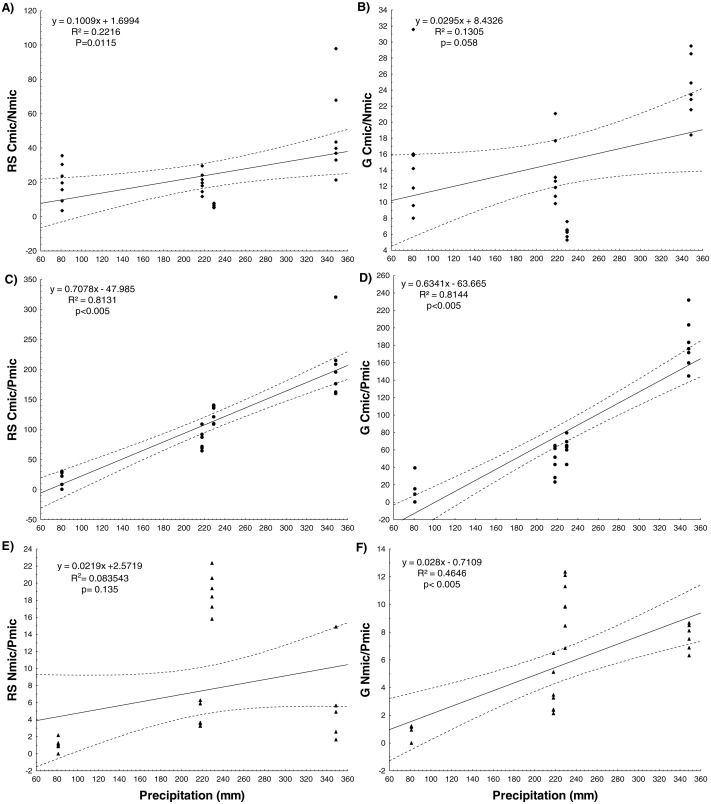
Simple linear regressions between the annual accumulated precipitation before the sampling date for four years and ratios of nutrients immobilized by microbial biomass for RS soil and G soil.

Significant positive correlations were observed between precipitation and immobilized nutrients within the microbial biomass (Cmic, Nmic, Pmic), in both soils. Moreover, significant positive correlations were detected between precipitation and the Cmic:Nmic and Cmic:Pmic ratios in the RS soil and the Cmic:Pmic and Nmic:Pmic ratios in the G soil. The slopes of the regression with Cmic and Nmic were higher in the G soil and lower in the RS soil (Figs. 1, 2 and Table S1).

**Figure 3:**
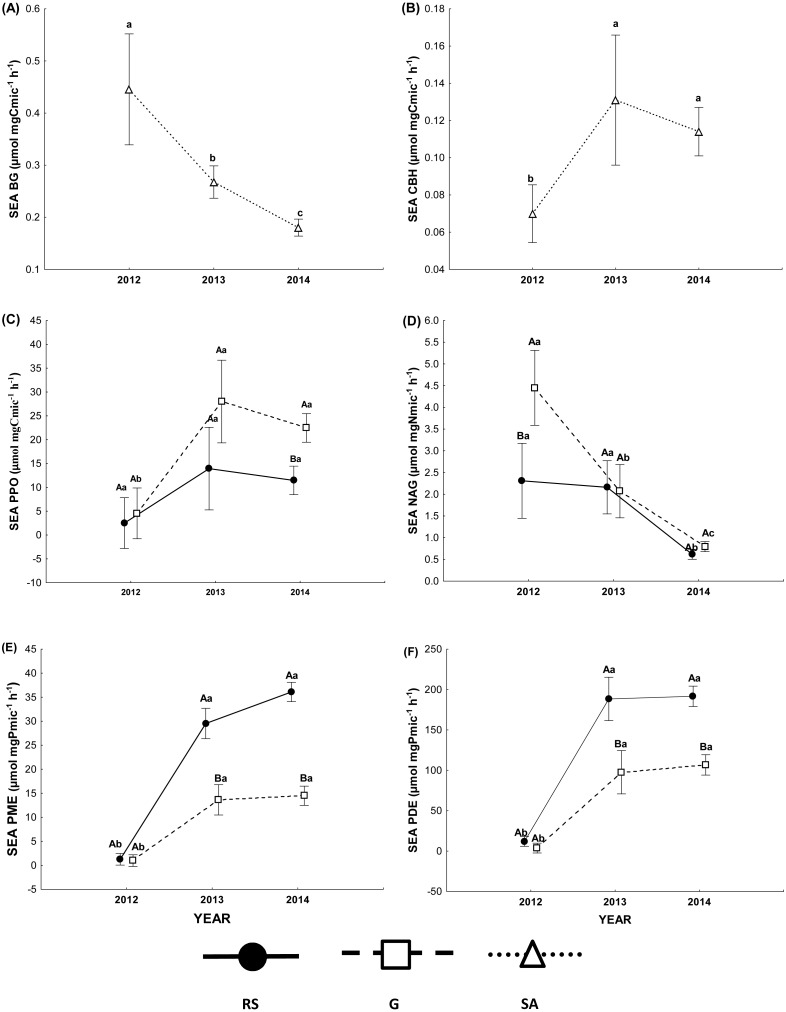
Specific enzymatic activity. (A) *β*-1,4-glucosidase (BG), (B) cellobiohydrolase (CBH), (C) polyphenol oxidase (PPO), (D) *β*-1,4-N-acetylglucosaminidase (NAG), (E) phosphomonoesterase (PME) and (F) phosphodiesterase (PDE) in the rosetophylous scrub (RS) and grassland (G) soils over three consecutive years (2012, 2013 and 2014) in the Cuatro Ciénegas Basin, Coahuila, Mexico. Different uppercase letters (A and B) indicate significantly different means (*P* < 0.05) between vegetation cover types (RS and G) within the same sampling year (2012, 2013 and 2014); whereas different lowercase letters (a, b and c) vertically indicate significantly different means (*P* < 0.05) among sampling dates within the same site.

The specificenzymatic activity of BG under both vegetation cover types was lower in the wet (2014) than in the dry year (2012; Fig. 3A, Table 4), while that of CBH in the dry year was lower than in both wet years (2013 and 2014), in both vegetation covers (Fig. 3B). The specific enzymatic activity of the PPO in the scrub soil did not differ among years, while the dry year (2012) had lower values than the wet years (2013 and 2014) in the G soil (Fig. 3C and Table 4). Furthermore, the G soil had higher specific PPO enzimatic activity than the RS soil in the wet year (2014). In contrast, the wet year (2014) had the lowest NAG specific enzymatic activity under both vegetation cover types, and the RS soil had lower values only in the dry year (2012; Fig. 3D). The specific enzymatic activity ofPME and PDE was similar, and the lowest values of specific enzymatic activity were in the driest year (2012). In the two wet years, the RS soil presented higher specific activities than the G soil (Figs. 3E and 3F).

### Soil bacterial composition

**Figure 4:**
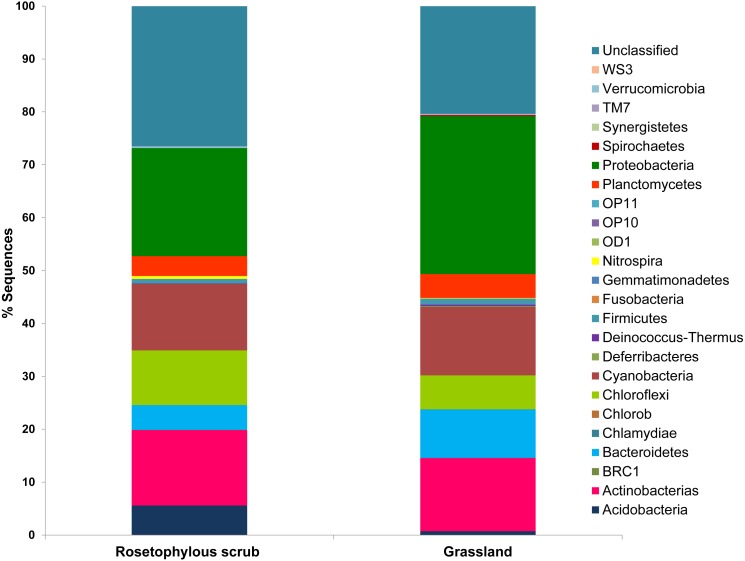
Taxonomic distribution of sequences obtained from Pyrosequencing of 16S rRNA tags of rosetophylous scrub and grassland soils during a wet year (2010).

Even at 97% similarity, a very high diversity was found, encompassing all the known phyla of bacteria but a very low diversity and abundance of Archaea. A total of 46,898 sequences were obtained for the RS soil and 9,979 for the G soil, comprising 24 phyla. We observed a high number of unclassified bacteria; 26% for the RS soil and 20% for the G soil (Fig. 4). In the two vegetation cover types, the Proteobacteria was the most abundant bacterial phylum, accounting for 20% in the RS soil and 30% in the G soil. Similarly, Actinobacteria was the second most dominant phylum in the RS soil and in the G soil, with an abundance of 14% in both soils. Interestingly, the Cyanobacteria was the third most dominant phylum, with 13% of abundance both soils, suggesting the importance of the desert crust in both sites. Other important phyla observed were: Chloroflexi (10%), Bacteroidetes (5%), Plantomycetes (4%), Firmicutes (4%), Nitrospira (1% in the RS and 0.5% in the G soils) and Acidobacteria (6% in RS and 0.8% in G; Fig. 4).

### Ecoenzymatic stoichiometry, homeostasis and threshold elemental ratios

In all of the model II regressions analyzed, there were no differences found in slopes between soils of the two vegetation cover types within sampling years (Figs. S1 and S2). To test the strength of stoichiometric homeostasis, we analyzed for associations between microbial biomass elemental ratios and those in the soil resources (Tapia-Torres et al., 2015a). In both soil vegetation cover types, the relationships between log C:N_R_ and log C:N_B_, and between log C:P_R_ and log C:P_B_ did not differ from zero (*p* > 0.05), regardless of year (Figs. S1 and S2); indicating strong community-level elemental homeostasis in the soil of both sites.

**Figure 5:**
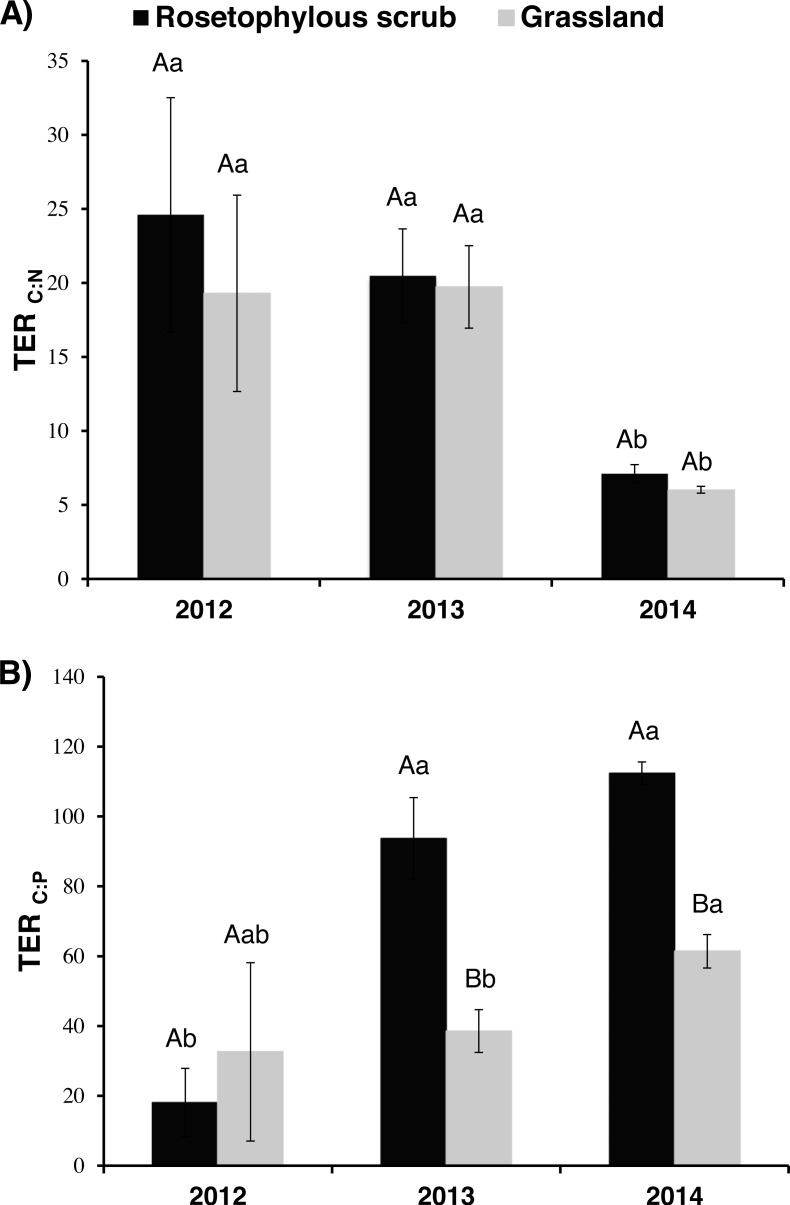
Threshold Elemental Ratio C:N and C:P (A and B, respectively) of the soil microbial community over three consecutive years (2012, 2013 and 2014).

Moreover, we used the parameters generated from the type II regressions using enzymatic data and microbial C:N:P stoichiometric values to estimate TER_C:N_ and TER_C:P_ values. The lowest TER_C:N_ values were observed in 2014 (wet year), but no differences were observed between 2012 and 2013, or even between vegetation cover types (RS and G) among study years (Fig. 5). The opposite was found for TER_C:P_, where we obtained the lowest value in the dry year (2012), but only in the RS soil. For the dry year (2012), no differences were observed between vegetation cover types, while we observed lower TER_C:P_ values in the G soil than in the RS soil for the wet years (2013 and 2014; Fig. 5).

## Discussion

### Reallocation of resources by the soil microbial community

Our first prediction, that the soil microbial community invests more energy in the production of ecoenzymes to acquire nutrients in sites of low resource availability, such as the RS soil, was confirmed. We observed that the RS soil showed a lower concentration of available P than the G soil in the three years studied and, consequently, the RS soil microbial community invested more energy in the acquisition of P (increased enzymatic activity of phosphomonoesterase and phosphodiesterase) than the G soil microbial community only during the two wet years. In contrast, the G soil had higher Cmic, Nmic and Pmic concentrations and lower enzymatic activity of phosphomonoesterase and phosphodiesterase than the RS soil during both wet years, which also supports our prediction (Fig. 1 and Table 3). These results suggest that the microbial community in the RS soil, with lower resource availability, must reduce growth as a result of: (1) the physiological cost associated with a low reallocation to P-rich ribosomal RNA, as suggested by the growth rate hypothesis (GRH) (Sinsabaugh & Follstad Shah, 2012; Sterner & Elser, 2002; Zechmeister-Boltenstern et al., 2015) and (2) the required investment of energy towards the acquisition of P in order to produce ecoenzymes (Evans & Wallenstein, 2012; Schimel, Balser & Wallenstein, 2007; Wallenstein & Hall, 2012). The microbial C:N:P ratio was greater in the RS soil (127:19:1) than in the G soil (63:10:1), suggesting that the microbial community in the former site is more P-constrained (Cleveland & Liptzin, 2007). The studied soils are characterized by low P availability and a high capacity for P occlusion within inorganic molecules, mainly by Ca-bound (Perroni et al., 2014). Therefore, the main source of available P is mineralization of organic P mediated by phosphatase activity (Waring, Weintraub & Sinsabaugh, 2014). Among organic P molecules, phosphodiester forms are the preferred substrate in P-limited ecosystems (Karl, 2014; Tapia-Torres et al., 2016), although phosphomonoester forms may also be an important source of available P in most soils (Turner, Mahieu & Condron, 2003). In our study sites, phosphodiesterase activity was almost ten times higher than that of phosphomonoesterase, mainly in the scrub soil, suggesting mineralization of phosphodiesters as the main source of soil available P. Several bacteria isolates from CCB soils prefer to grow in DNA as a P source, associated with phosphodiesterase activity (Tapia-Torres et al., 2016). We suggest that the main P source in sites with low nutrient availability, such as the RS soil, is recycling of the organic molecules that are the product of cellular lysis.

However, the G soil had higher enzymatic activity of polyphenol oxidase in the wet year 2014 than was the case in the RS soil. This result is consistent with other studies (Sinsabaugh, 2010; Sinsabaugh & Follstad Shah, 2011), which have reported that polyphenol oxidase activity does not present the same behavior as the *β*-1,4-glucosidase and other hydrolases that degrade labile C. Microbial community size begins to be limited by the availability of labile C, which produces a change in the microbial community composition towards microbial guilds with lower growth rates (low concentration of Cmic), but with the capacity to produce polyphenol oxidase to break down structurally complex molecules and obtain C (Moorhead & Sinsabaugh, 2006). This situation is comparable to the conditions of the G soil in the wet year 2014, where the microbial community was required to cleave lignin in order to maintain its growth rate.

Furthermore, the differences in soil nutrient dynamics between both sites can be strongly affected by soil microbial composition. While analyses of soil bacterial composition were only determined for 2011, this year presented the highest soil water availability and also showed higher concentrations of Cmic, Nmic, and Pmic than in the other studied years. Several studies (Nemergut et al., 2010; Philippot et al., 2009) have reported that heterotrophic decomposition depends on the relative abundance of specific taxa because different species process organic matter at different rates, even under similar soil conditions. The G soil had a higher proportion of Proteobacterias, Actinobacterias and Bacteroidetes than the RS soil, and some species of these taxa have the capacity to produce *β*-glucosidase (BG) (Moreno et al., 2013), cellobiohydrolase (CBH), poliphenoloxidase (PPO) (Kirk & Farrell, 1987), glucanases and glycosidases (Xie et al., 2007), which act to cleave C molecules.

In contrast, the scrub soil had higher proportion of Acidobaterias and Firmicutes, including species with the capacity for producing enzymes for P mineralization (Koch et al., 2008; Tan et al., 2013). Moreover, the acidobacterias of the RS soil could contribute to the release of unavailable P through organic acid release (Tan et al., 2013) and, together with Firmicutes, can mineralize P via the production of phosphatases, as has been observed in isolates of acidobacterias from substrates with low C concentrations (Koch et al., 2008; Tan et al., 2013). Chloroflexi was present in a higher proportion in the RS than in the G soil, but both soils had a similar proportion of Cyanobacteria suggesting that the amount of microbial desert crust is similar in both sites. Both phyla are facultative autotrophic bacteria (Smith, 1983) and therefore have the capacity to fix atmospheric C and to produce ecoenzymes for depolymerization and mineralization of C (Berg et al., 2010; Mitsui et al., 1986; Smith, 1983). The Cyanobacteria also have the capacity to fix atmospheric N. Fixation in the microbial biomass of C and N by these taxa could represent an important input of both nutrients to the soil (Mitsui et al., 1986; Smith, 1983). Wallenstein & Hall (2012) proposed that sites limited by nutrients are more vulnerable to rainfall variability, because the microbial community must invest energy in nutrient acquisition, thus reducing its capacity for adaptation required by fluctuation in water availability. We proposed that sites with low resources availability, such as the RS soil, could be thus more vulnerable to annual precipitation variability.

### Resilience in the face of precipitation changes

Our second prediction, that the microbial community will be more vulnerable to variability in precipitation in the site with lower soil resources (RS), was not confirmed because the soil community was resilient to soil P coinstrains by ecoenzyme upregulation during times of adequate moisture. In both vegetation cover types, nutrient availability increased with increased precipitation. The correlation between precipitation and the Cmic, Nmic and Pmic, indicate that a higher amount of rainfall favored the microbial immobilization of these nutrients under both vegetation cover types. Nevertheless, compared to the RS soil, the G soil showed steeper slopes in regressions between the precipitation and the concentrations of Nmic and ratios of Cmic:Pmic and Nmic:Pmic (Table S1), suggesting that the microbial community of the grassland soil has the ability to immobilize more N within its microbial biomass and more rapidly than the microbial community of the RS soil. Positive correlations between Cmic and rainfall have been reported for an oak forest (Baldrian et al., 2010) and a semiarid grassland (Zhou et al., 2013), but a correlation between precipitation with Nmic and Pmic concentrations has not hitherto been reported for natural ecosystems.

Furthermore, in the soil community homeostasis analyses, the relationships between log C:N_R_ and log C:N_B_, and between log C:P_R_ and log C:P_B_ in the G and the RS soils had slopes that did not differ significantly from zero (Figs. S1 and S2), suggesting that the soil microbial communities adjust physiologically (Sinsabaugh & Follstad Shah, 2012) to processing low N and P resources in order to cope with the nutrient limitation, particularly in dry years. Our data also suggest that these physiological adjustments occurred differently in the soil microbial communities of the two vegetation covers and was related to both precipitation quantity and nutrient availability.

Our results show how values of TERC:N and TER_C:P_ may shift with respect to variation in annual rainfall and different vegetation cover. The estimated TER_C:N_ was lower in the wet year for both sites, indicating greater sensitivity to N limitation due to the rapid growth of the microbial community produced by the water availability. For TER_C:P_, we observed site-specific differences. The TER_C:P_ was higher in the RS soil than in the G soil for 2013 and 2014, indicating a greater sensitivity of the microbial community to P limitation in the G soil. However, in order to determine the nutritional limitations of the microbial community, we also compared the estimated TER values and the C:N or C:P ratios of the organic matter. If the C:N or C:P ratio of the organic matter being consumed is greater than the TER for that element, this would suggest nutrient limitation (Sterner & Elser, 2002). We observed P limitation in both soils, regardless of year (C:P > TER_C:P_; *p* < 0.05) and N limitation in the G soil in the wet year (C:N = 11.3 and TER_C:N_ = 6; *p* = 0.002). Our results for the dry year (2012) showed that the ecoenzymatic activities associated with C and P acquisition were lowest in the RS and G soils. Values for TERC:N and TER_C:P_ were similar between the RS and G soils, suggesting that both sites may be vulnerable to drought. However, with the increase of the annual precipitation (years 2013 and 2014), the G soil microbial community requires more P and N to meet its metabolic demands and it makes metabolic adjustments in order to maintain its growth which makes it more susceptible or sensitive to resource limitation. Similarly, increased ecoenzyme activities associated with P acquisition and elevated TER_C:P_ values when the water is not limiting (2013 and 2014) suggest that the RS soil microbial community is well adapted to acquire P resources via ecoenzyme upregulation post drought.

We suggested that, under the scenario proposed by Global Climate Change models for desert ecosystems that predict reduced annual precipitation and increased rainfall variability, the microbial community from both sites could be vulnerable to drought events, but the RS soil microbial communities can make adjustments in order to obtain nutrients in wet years, suggesting that this community is resilient post drought.

## Conclusion

Soil communities of both sites (RS and G) may be vulnerable to drought. However, the community at the site with lower resources (RS) may have evolved adaptations, such as rapid ecoenzymatic upregulation, under chronic P limitation. This adaptation confers greater resilience within the community to respond to precipitation events post drought. Under the Global Climate Change scenarios for desert ecosystems that predict reduced annual precipitation and an increased intensity and frequency of torrential rains and drought events, soil microbial communities within both sites could be vulnerable to drought through the combination of C and P co-limitation and reallocation of energy and nutrient resources to physiological acclimatization strategies in order to survive.

##  Supplemental Information

10.7717/peerj.4007/supp-1 Figure S1 Simple linear regressions between C:N microbial biomass and C:N soil resources Regression slope (mx), correlation coefficient (*R*^2^) and statistical significance (*P* < 0.05) are shown for the rosetophylous scrub (RS) and the grassland (G) soils for three years (2012, 2013 and 2014) in Cuatro Ciénegas Basin, Coahuila Mexico. (A) RS soil at 2012, (B) RS soil at 2013, (C) RS soil at 2014, (D) G soil at 2012, (E) G soil at 2013, and (F) G soil at 2014.

10.7717/peerj.4007/supp-2 Figure S2 Simple linear regressions between C:P microbial biomass and C:P soil resources Regression slope (mx), correlation coefficient (*R*^2^) and statistical significance (*P* < 0.05) are shown for the rosetophylous scrub (RS) and the grassland (G) soils for three years (2012, 2013 and 2014) in Cuatro Ciénegas Basin, Coahuila Mexico.

10.7717/peerj.4007/supp-3 Data S1 Raw data

10.7717/peerj.4007/supp-4 Table S1 Simple linear regression between annual rainfall and nutrient concentrations within microbial biomass Regression slope (mx), correlation coefficient (*R*^2^) and statistical significance (*P* < 0.05) are shown between C, N, and P in microbial biomass quantified in the rosetophylous scrub (RS) and the grassland (G) soils and the annual accumulated precipitation for four years (2010, 2012, 2013 and 2014) in Cuatro Ciénegas Basin, Coahuila Mexico.
