# Supplementary figures and images for: The response of soil microbial communities to variation in annual precipitation depends on soil nutritional status in an oligotrophic desert
Source: PeerJ. 2017 Nov 9;5:e4007. doi: 10.7717/peerj.4007 (PMC5682101; doi:10.7717/peerj.4007)

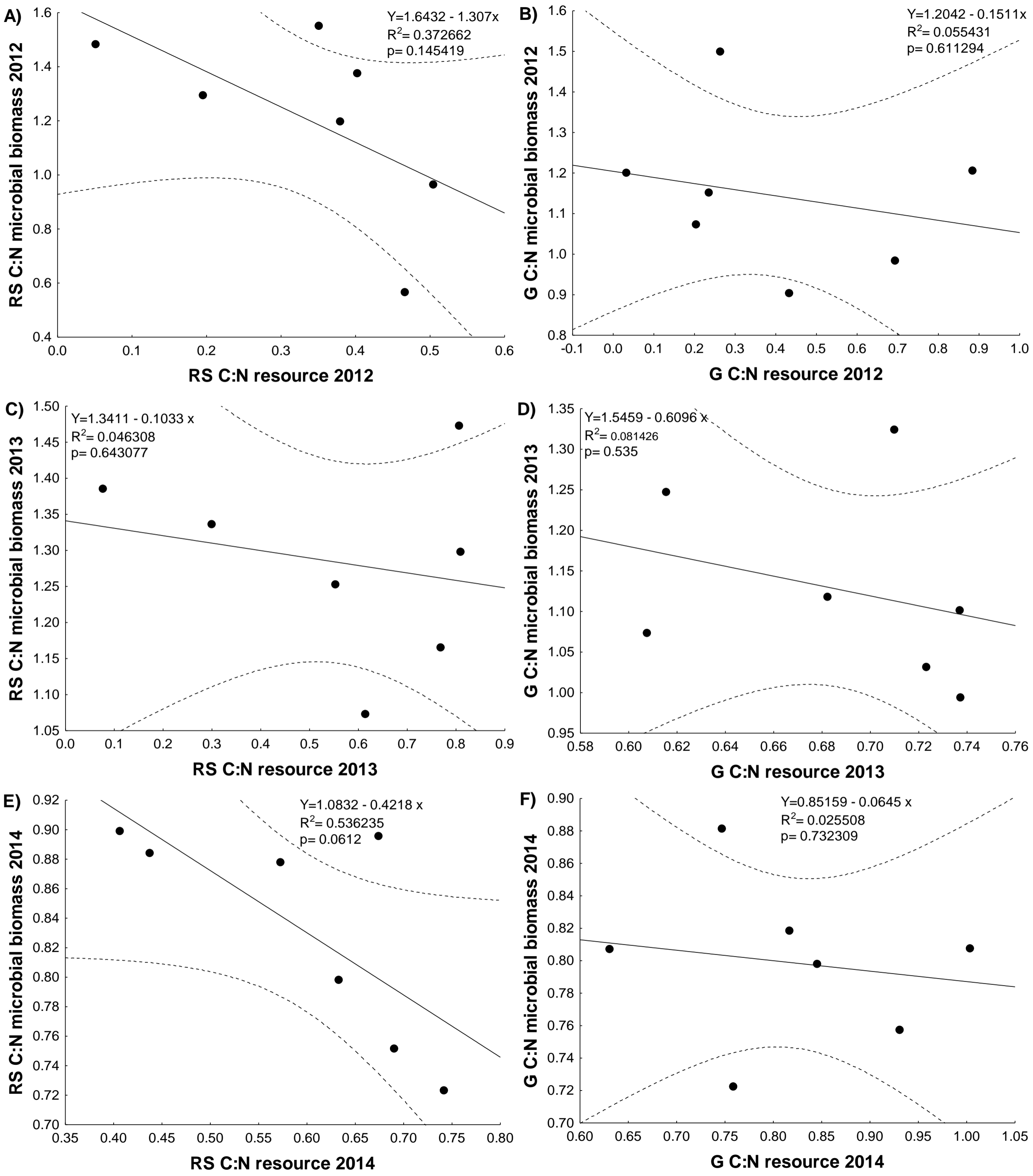

Supplement: Figure S1 — Regression slope (mx), correlation coefficient (R2) and statistical significance (P < 0.05) are shown for the rosetophylous scrub (RS) and the grassland (G) soils for three years (2012, 2013 and 2014) in Cuatro Ciénegas Basin, Coahuila Mexico. (A) RS soil at 2012, (B) RS soil at 2013, (C) RS soil at 2014, (D) G soil at 2012, (E) G soil at 2013, and (F) G soil at 2014. [file peerj-05-4007-s001.pdf]

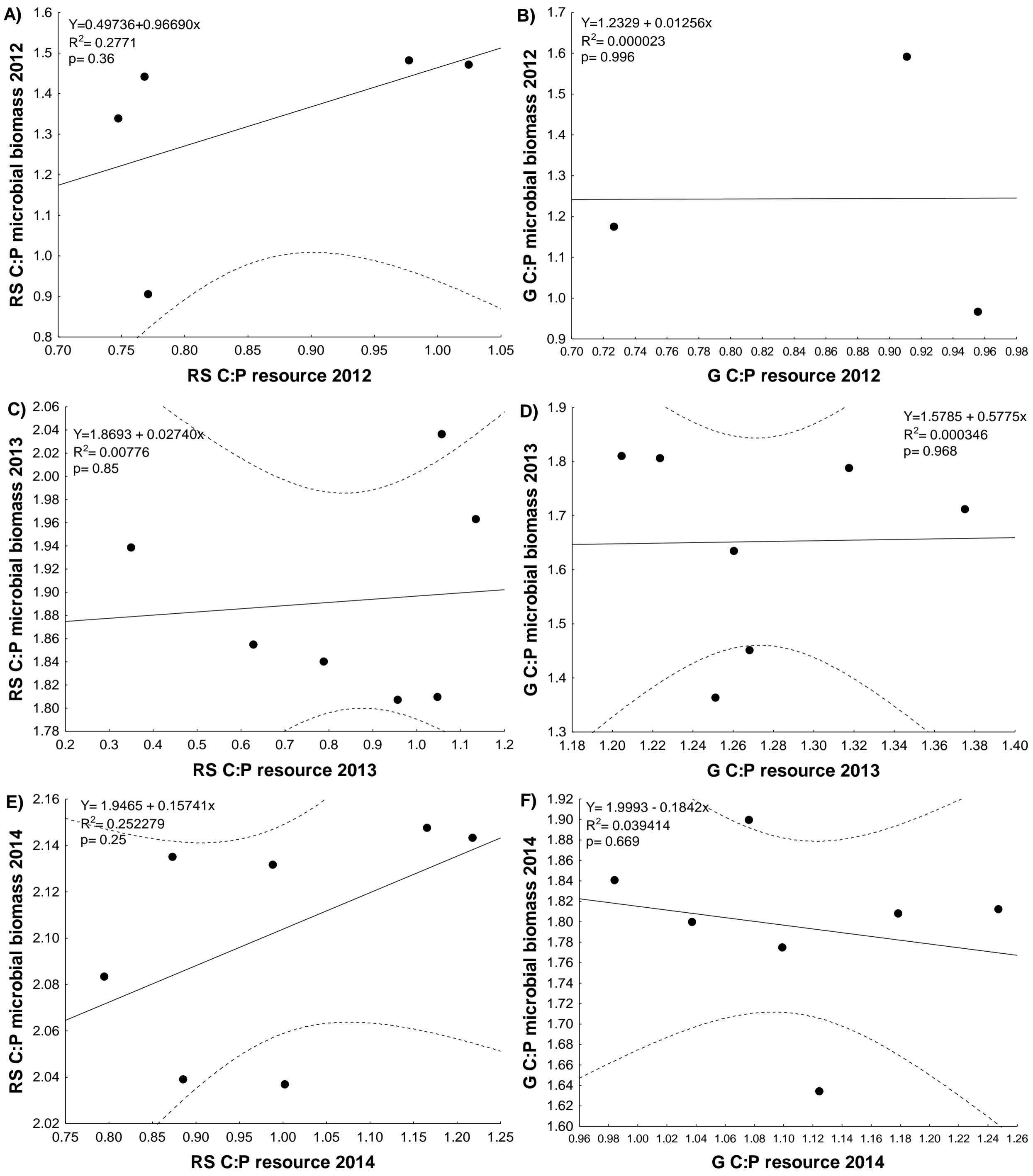

Supplement: Figure S2 — Regression slope (mx), correlation coefficient (R2) and statistical significance (P < 0.05) are shown for the rosetophylous scrub (RS) and the grassland (G) soils for three years (2012, 2013 and 2014) in Cuatro Ciénegas Basin, Coahuila Mexico. [file peerj-05-4007-s002.pdf]
