# Supplementary material for: The response of soil microbial communities to variation in annual precipitation depends on soil nutritional status in an oligotrophic desert
Source: PeerJ. 2017 Nov 9;5:e4007. doi: 10.7717/peerj.4007 (PMC5682101; doi:10.7717/peerj.4007)
Supplement: Table S1 — Regression slope (mx), correlation coefficient (R2) and statistical significance (P < 0.05) are shown between C, N, and P in microbial biomass quantified in the rosetophylous scrub (RS) and the grassland (G) soils and the annual accumulated precipitation for four years (2010, 2012, 2013 and 2014) in Cuatro Ciénegas Basin, Coahuila Mexico. [file peerj-05-4007-s004.pdf]

Table S1. Simple linear regression between annual rainfall and nutrient concentrations within microbial biomass. Regression slope (mx), correlation coefficient (R^2) and statistical significance ($P<0.05$) are shown between C, N, and P in microbial biomass quantified in the rosetophylous scrub (RS) and the grassland (G) soils and the annual accumulated precipitation for four years (2010, 2012, 2013 and 2014) in Cuatro Ciénegas Basin, Coahuila Mexico.

Soil Variable	RS		G	
	Mx	R^2 (p)	mx	R^2 (p)
Cmic	2.96	0.77 (<0.0001)	0.01	0.83 (<0.0001)
Nmic	0.07	0.20 (0.02)	0.15	0.43 (0.0001)
Pmic	0.01	0.23 (0.009)	0.01	0.29 (0.009)
C:Nmic	0.07	0.22 (0.01)	0.03	0.13 (0.058)
C:Pmic	0.07	0.81 (<0.0001)	0.63	0.81 (<0.0001)
N:Pmic	0.02	0.08 (0.13)	0.03	0.46 (<0.0001)

Cmic: microbial carbon; Nmic: microbial nitrogen; Pmic: microbial phosphorus
